# ﻿*Scutellaria cavicola* (Lamiaceae), a new cave-dwelling species from Northwest Guangxi, China

**DOI:** 10.3897/phytokeys.262.158923

**Published:** 2025-09-12

**Authors:** Chi Xiong, Zi-Yu Wei, Rui-Ning Liu, Feng Chen, Xiao-Ying Fu, Yi-Chen Wang, Long-Fei Fu

**Affiliations:** 1 Guangxi Key Laboratory of Plant Conservation and Restoration Ecology in Karst Terrain, Guangxi Institute of Botany, Guangxi Zhuang Autonomous Region and Chinese Academy of Sciences, Guilin, 541006, Guangxi, China Guangxi Zhuang Autonomous Region and Chinese Academy of Sciences Guilin China; 2 School of Chinese Pharmacy, Beijing University of Chinese Medicine, Beijing 100029, China Beijing University of Chinese Medicine Beijing China; 3 Forestry College, Guizhou University, Guiyang, 550025, China Guizhou University Guiyang China; 4 Chongqing Museum of Natural History, Beibei, 400799, Chongqing, China Chongqing Museum of Natural History Chongqing China; 5 College of Life Sciences, Guangxi Normal University, Guilin 541006, Guangxi, China Guangxi Normal University Guilin China; 6 Hangzhou Botanical Garden, Hangzhou, 310013, China Hangzhou Botanical Garden Hangzhou China

**Keywords:** Flora of Guangxi, Karst cave, Lingyun County, phylogeny, *Scutellaria tsinyunensis*, taxonomy

## Abstract

*Scutellaria cavicola* C.Xiong, Z.Y.Wei & L.F.Fu, a new cave-dwelling species discovered in Lingyun County, Guangxi, China, was described and illustrated. The new species morphologically resembles *S. tsinyunensis* in having ligneous stems, stiffly papery leaves, undulate leaf margins. However, it can be clearly distinguished from *S. tsinyunensis* in the height of plants, the shape of leaf blades, the length of petiole and racemes, the size of leaves and nutlets. The results of molecular phylogenetic analysis, based on nuclear ribosome internal transcribed spacer (ITS) and external transcribed spacers (ETS) of 43 *Scutellaria* species, showed that *S. cavicola* is closely related to *S. tsinyunensis*. Notably, according to the IUCN Red List Categories and Criteria, the new species is assigned to the Critically Endangered (CR) category.

## ﻿Introduction

*Scutellaria* L. is one of the most species-rich genera within Lamiaceae, comprising more than 476 species (POWO 2025). The genus exhibits a broad distribution across tropical mountainous regions and temperate zones. Notably, the Iran-Turan region, particularly Central Asia and the Iranian Plateau, is recognised as the primary centre of diversity for *Scutellaria* (Paton 1990). In comparison, China represents a major centre of distribution, with approximately 100 species and 25 varieties recorded (Xiang 2016; Zhao et al. 2017), including 11 species and five varieties documented in Guangxi (Wei 2023). Species of *Scutellaria* are annual or perennial herbs, or subshrubs (Wu and Li 1977). The genus is characterised by its distinctive calyx, which possesses two undivided lips and an appendage that folds into an upright, sail-like structure on the upper lip, referred to as the scutellum, from which the genus derives its name. In some cases, this structure appears as an appendage intumescence rather than a scutellum (Paton 1990). *Scutellaria* is renowned for its medicinal value, numerous species, such as *S. baicalensis* Georgi, *S. barbata* D. Don and *S. indica* L., being known for their anti-cancer, anti-inflammatory and antioxidant properties (Miyaichi et al. 1987, 1989; Sato et al. 2000; Kudo et al. 2017. These species have been cultivated and utilised in traditional Chinese medicine for over 2,000 years (Wang et al. 2024).

In October 2023, a botanical survey of vascular plants in the karst caves of Linyun County, located in North-western Guangxi, led to the discovery of an unknown small herb bearing fruits at the entrance of a cave. The plant was identified as belonging to the genus *Scutellaria*, based on its 4-angled stems, opposite leaves and scutellar epicalyx lobes. A follow-up visit in August 2024 yielded flowering specimens, which were carefully dissected and photographed. A thorough examination of herbarium specimens and a comprehensive review of the relevant literature (Wu and Li 1977; Li and Hedge 1994; Zhao et al. 2017; Ding et al. 2019; Tan et al. 2022; Wei 2023; Zhang et al. 2024) confirmed that the plant represents a new species of *Scutellaria*. In addition, we investigated the phylogenetic position of the new taxon, which is formally described and illustrated herein.

## ﻿Materials and methods

### ﻿Morphological analyses

Specimens of the newly-identified species were collected during a field expedition to Linyun County, Northwest Guangxi in 2024. Photographs were taken using a Nikon D7200 digital camera (Japan). All morphological characters were carefully examined using both field-collected and herbarium specimens under an Olympus SZX16 binocular microscope. Voucher specimens were deposited at IBK and KUN (herbarium acronyms follow Thiers (2025)). Nutlet morphology was observed using a camera (Canon R5) fitted with a high-magnification objective lens (Mitutoyo). At least ten nutlets were used to determine their size and surface ornamentation. Additional specimens of closely-related species housed at BJFC, KUN, PE and SM were examined through the Chinese Virtual Herbarium (https://www.cvh.ac.cn/). Morphological terminology follows Li and Hedge (1994) and Beentje (2016).

### ﻿Genomic DNA extraction and sequencing

Total genomic DNA was extracted from silica-dried leaves using a modified CTAB method (Chen et al. 2014). The DNA samples were sent to Majorbio (http://www.majorbio.com/) for library construction and next-generation sequencing. A paired-end library with a 350 bp was constructed and sequencing was performed using the Illumina HiSeq4000 platform. Approximately 1 Gb of raw reads was generated and subsequently filtered using the FASTX-Toolkit to remove adapter and low-quality reads (http://hannonlab.cshl.edu/fastx_toolkit/download.html). Clean reads were assembled using GetOrganelle v.1.7.7.0 (Jin et al. 2020) to recover ribosomal genome sequences. ITS regions were extracted using ITSx v.1.1.3 (Bengtsson-Palme et al. 2013), while ETS sequences were obtained from the upstream region of the 18S gene identified through ITSx positional data.

### ﻿Phylogenetic analyses

To determine the phylogenetic placement of this species, we extracted the nuclear ribosomal ITS and ETS from the assembled genome. The resulting sequences have been deposited in GenBank under accession numbers PV501071 and PV506383. Additional sequences from *Scutellaria* and related taxa were downloaded from GenBank based on previous studies (Zhao et al. 2017; Tan et al. 2022) (see Table 1 for details). This final dataset included 47 accessions representing 45 taxa, with 43 taxa belonging to *Scutellaria* as the ingroup and one species each from *Holmskioldia* Retz. and *Tinnea* Kotschy ex Hook.f. as outgroups.

**Table 1.** Voucher information for phylogenetic analyses and GenBank accession numbers.

Taxa	Voucher/Herbarium barcode	Location	ITS	ETS
*Scutellaria alpina*	Liao PC, s.n.	Europe alpine region	MF193544	MF193591
*Scutellaria axilliflora*	Hu GX, H144 (KUN)	Fujian, China	MF193529	MF193575
*Scutellaria baicalensis*	Li DZ et al., 0513 (KUN)	Liaoning, China	MF193525	MF193571
*Scutellaria barbata*	Xiang CL, 282 (KUN)	Beijing, China	MF193539	MF193585
*Scutellaria calcarata*	Shui YM et al., Z-03343396 (KUN)	Yunnan, China	MF193512	MF193558
*Scutellaria cavicola*	Xiong C et al., XC24010 (IBK)	Guangxi, China	PV501071	PV506383
*Scutellaria dependens*	Anonymous, 316	Fujinomiya, Japan	MF193538	MF193584
*Scutellaria diffusa*	Wang ZH, s.n (KUN)	Germany	MF193541	MF193587
*Scutellaria discolor*	Xiang CL et al., 438 (KUN)	Yunnan, China	MF193504	MF193550
*Scutellaria franchetiana*	Xiang CL, 287 (KUN)	Yunnan, China	MF193532	MF193578
*Scutellaria galericulata*	M-14212	Iran	MF193535	MF193581
*Scutellaria hainanensis*	Jiang L et al., 398 (KUN)	Hainan, China	MF193505	MF193551
*Scutellaria hunanensis*	Hu GX, H96 (KUN)	Hunan, China	MF193531	MF193577
*Scutellaria indica*	Peng H, s.n (KUN)	Hong kong, China	MF193513	MF193559
*Scutellaria jishouensis*	Chen GX et al., DHK-20210418	Hunan, China	OM287561	OM307410
*Scutellaria kingiana*	Zhang JW et al., ZJW-3890 (KUN)	Xizang, China	MF193542	MF193588
*Scutellaria likiangensis*	Xiang CL et al., 373 (KUN)	Yunnan, China	MF193524	MF193570
*Scutellaria macrodonta*	Zhao F et al., 2015-006 (KUN)	Beijing, China	MF193523	MF193569
*Scutellaria mairei*	Shui YM et al., 66205 (KUN)	Yunnan, China	MF193516	MF193562
*Scutellaria nepetifolia*	TUH-27605 (THU)	Iran	MF193545	MF193592
*Scutellaria nuristanica*	M-32142	Iran	–	MF193589
*Scutellaria obtusifolia*	Chen YP et al., EM202 (KUN)	Sichuan, China	MF193508	MF193554
*Scutellaria orthocalyx*	Xiang CL, 185 (KUN)	Yunnan, China	MF193527	MF193573
*Scutellaria platystegia*	TUH-7697(THU)	Iran	MF193546	MF193593
*Scutellaria regeliana*	Jiang L, 149 (KUN)	Neimenggu, China	MF193536	MF193582
*Scutellaria scordifolia*	Yu WT et al., 2822 (KUN)	Qinhai, China	MF193540	MF193586
*Scutellaria sessilifolia* 1	Xiang CL, 341 (KUN)	Chongqing, China	MF193533	MF193579
*Scutellaria sessilifolia* 2	Peng H et al., 117 (KUN)	Sichuan, China	MF193534	MF193580
*Scutellaria shweliensis*	Zhao F et al., ZF0068 (KUN)	Yunnan, China	MF193530	MF193576
*Scutellaria sichourensis*	Xiang CL et al., 566 (KUN)	Yunnan, China	MF193509	MF193555
*Scutellaria stocksii*	M-30348	Iran	MF193543	MF193590
*Scutellaria subintegra*	Chen YP, EM223 (KUN)	Guangxi, China	MF193528	MF193574
*Scutellaria supina*	Liu B et al., CPG28095 (PE)	Xinjiang, China	MF193547	MF193594
*Scutellaria taiwanensis*	Liao PC, s.n. (KUN)	Taiwan, China	MF193515	MF193561
*Scutellaria tapintzeensis*	Cai J et al., 15cs11358 (KUN)	Yunnan, China	MF193518	MF193564
*Scutellaria tenax*	Peng H et al., 2012-017 (KUN)	Guizhou, China	MF193517	MF193563
*Scutellaria tenera*	Chen YP et al., EM187 (KUN)	Jiangxi, China	MF193522	MF193568
*Scutellaria teniana*	Xiang CL et al., 288 (KUN)	Yunnan, China	MF193520	MF193566
*Scutellaria tsinyunensis*	Xiong C et al., XC24060 (IBK)	Chongqing, China	PV501072	PV506384
*Scutellaria viscidula*	Zhao F, 2015-009 (KUN)	Hebei, China	MF193526	MF193572
*Scutellaria wenshanensis*	Zhao F et al., 008 (KUN)	Yunnan, China	MF193510	MF193556
*Scutellaria wuana*	Xiang CL et al., 1200 (KUN)	Sichuan, China	MF193521	MF193567
*Scutellaria yangbiensis*	Liu ED et al., 2238 (KUN)	Yunnan, China	MF193511	MF193557
Scutellaria yunnanensis var. cuneata	Xiang CL et al., 574 (KUN)	Yunnan, China	MF193507	MF193553
*Scutellaria yunnanensis*	Liu Ed et al., 3037 (KUN)	Yunnan, China	MF193506	MF193552
*Holmskioldia sanguinea*	Anonymous, 209	Guandong, China	MF193548	MF193595
*Tinnea rhodesiana*	Gary Stafford, GIS-359 (KUN)	South Africa	MF193549	MF193596

All sequences were aligned using MAFFT and aligned ITS and ETS regions were concatenated using Phylosuite v.1.2.3 (Katoh and Standley 2013; Zhang et al. 2020). The best-substitution model (GTR+F+I+G4) was selected using ModelFinder (Kalyaanamoorthy et al. 2017), with the corrected Akaike Information Criterion (AICc). Bayesian Inference (BI) analysis was performed in MrBayes with Phylosuite v.1.2.3 (Ronquist et al. 2012; Zhang et al. 2020). The Markov chains were run for 1,000,000 generations, with sampling every 1,000 generations and discarding the first 25% as burn-in. The Maximum Likelihood (ML) analysiswas conducted using IQ-TREE with 1,000 bootstrap replicates in Phylosuite v.1.2.3 (Guindon et al. 2010; Minh et al. 2013; Nguyen et al. 2015; Zhang et al. 2020).

## ﻿Results

**Figure 1.**
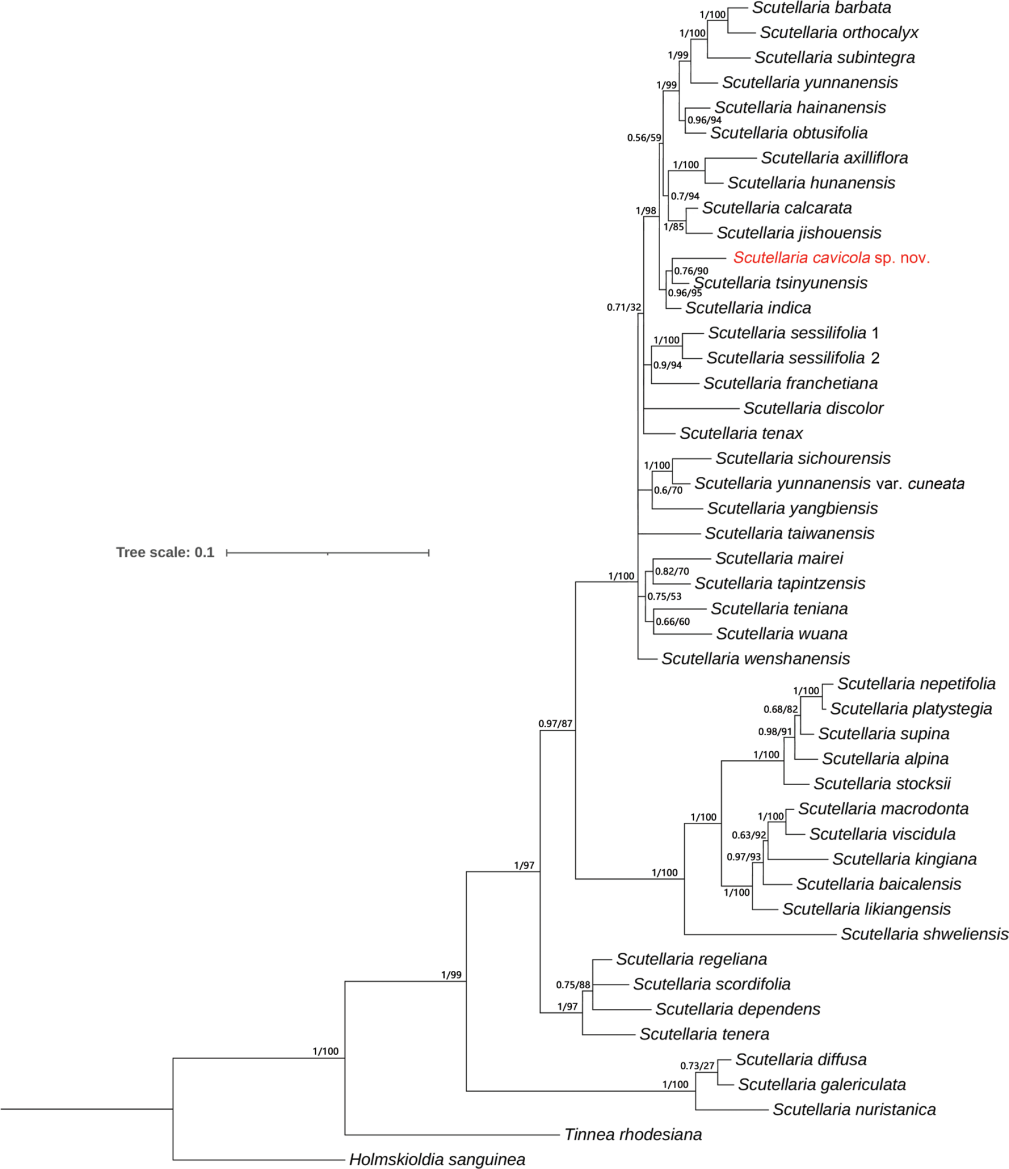
Bayesian tree from analysis of combined ITS and ETS data of 43 species of *Scutellaria*. The bootstrap values (BS) of ML and posterior probabilities (PP) of BI are listed at each node. The new species is highlighted in red.

The aligned ITS and ETS were 543 bp and 423 bp, respectively, yielding a concatenated alignment of 966 bp. The resulting phylogenetic tree (Fig. 1) was largely congruent with previous studies (Zhao et al. 2017; Tan et al. 2022). The new species formed a monophyletic clade with *S. tsinyunensis* (PP/BS = 0.76/90), which was resolved as sister to *S. indica* L. with strong support (PP/BS = 0.96/95) (Fig. 1).

## ﻿Taxonomic treatment

### ﻿ Scutellaria cavicola

Taxon classificationPlantaeLamialesLamiaceae

C.Xiong, Z.Y.Wei & L.F.Fu sp. nov.

DEE15574-35F0-57B8-AFF2-00A0429BD600

urn:lsid:ipni.org:names:77369127-1

Figs 2, 3

#### Diagnosis.

*Scutellaria cavicola* belongs to Scutellaria Ser. Javanicae C. Y. Wu (Wu and Li 1977) and exhibits morphological similarities to *S. tsinyunensis* C.Y. Wu & S. Chow (Wu and Li 1977) (Fig. 4). Both species are characterised by certain shared traits, including ligneous stems, leathery or stiffly papery leaves, undulate leaf margins. However, *S. cavicola* differs from *S. tsinyunensis* by its shorter stems (15–30 cm tall vs. 30–60 cm), lamina ovate to triangular-ovate, 14.5–23 × 8.5–14.5 mm (vs. ovate-lanceolate to lanceolate, 40–80 × 15–35 mm), petiole 3–7.5 mm long (vs. sessile or short, 0–4 mm), racemes 1–5 cm long and 3–10 flowers (vs. 8–10 cm, up to 30 flowers). A more detailed comparison of the two species is provided in Table 2.

**Table 2.** Comparative morphology of *Scutellaria cavicola* to *S. tsinyunensis*.

Characters	*S. cavicola*	*S. tsinyunensis*
height	15–30 cm	30–60 cm
leaf blades	ovate to triangular-ovate, 14.5–23 × 8.5–14.5 mm, base broadly cuneate, rounded to truncate, apex obtuse to acute	ovate-lanceolate to lanceolate, 40–80 × 15–35 mm, base roundish to shallowly cordate, apex caudate to caudate-acuminate
petiole	3–7.5 mm	sessile or short, 0–4 mm
racemes	1–5 cm, 3–10 flowers	8–10 cm, up to 30 flowers
nutlets	1.22–1.47 × 0.79–0.98 mm	ca. 2.8 × 1.6 mm
flowering time	August to October	April to May
distribution	Linyun, NW. Guangxi	Beibei & Jiangjin, Chongqing

**Figure 2.**
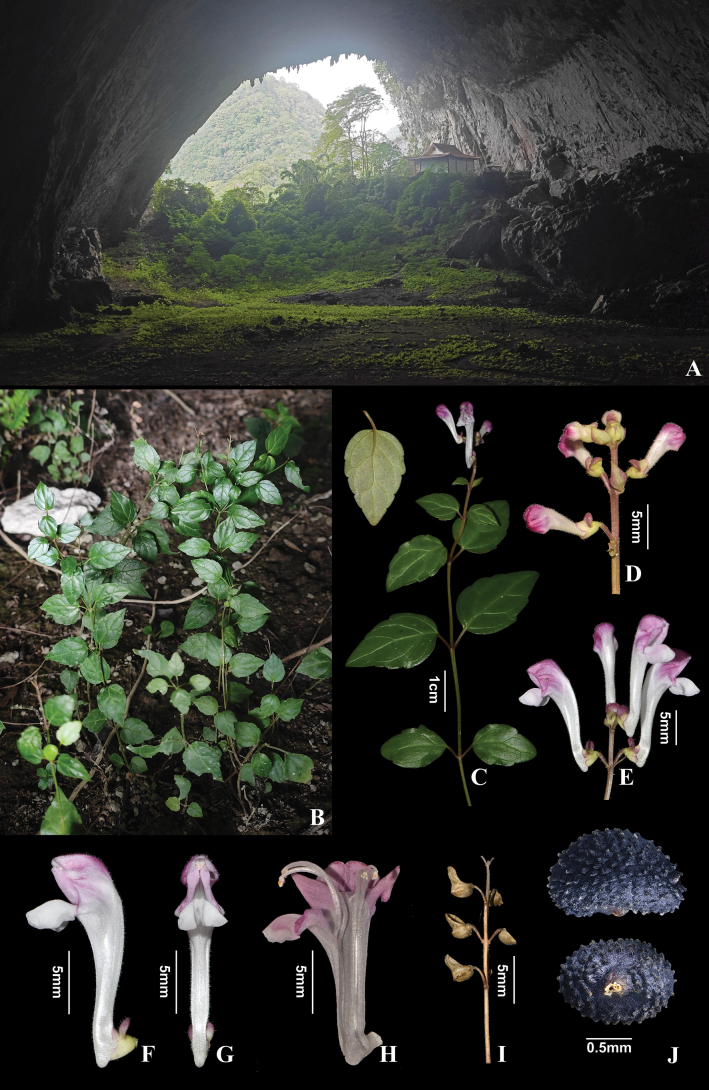
*Scutellaria cavicola* C.Xiong, Z.Y.Wei & L.F.Fu, sp. nov. A, B. Habitat; C. Flowering plant; D. Young inflorescence; E. Mature inflorescence; F. Flower (side view); G. Flower (front view); H. Dissection of a flower; I. Fruiting calyces; J. Nutlets.

**Figure 3.**
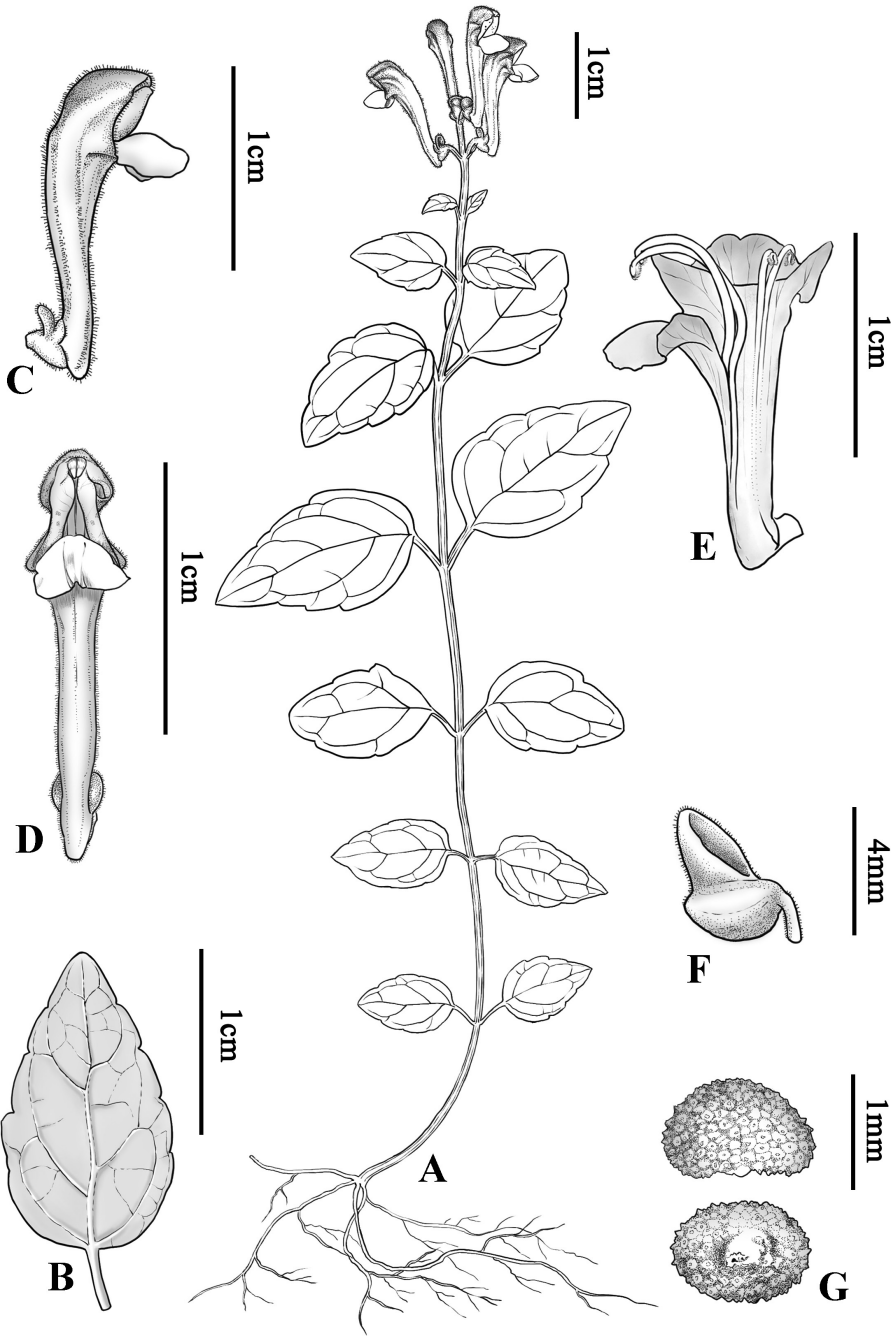
*Scutellaria cavicola* C.Xiong, Z.Y.Wei & L.F.Fu, sp. nov. A. Habit; B. Leaf blade; C. Flower (side view); D. Flower (front view); E. Corolla anatomy; F. Scutum; G. Nutlets.

**Figure 4.**
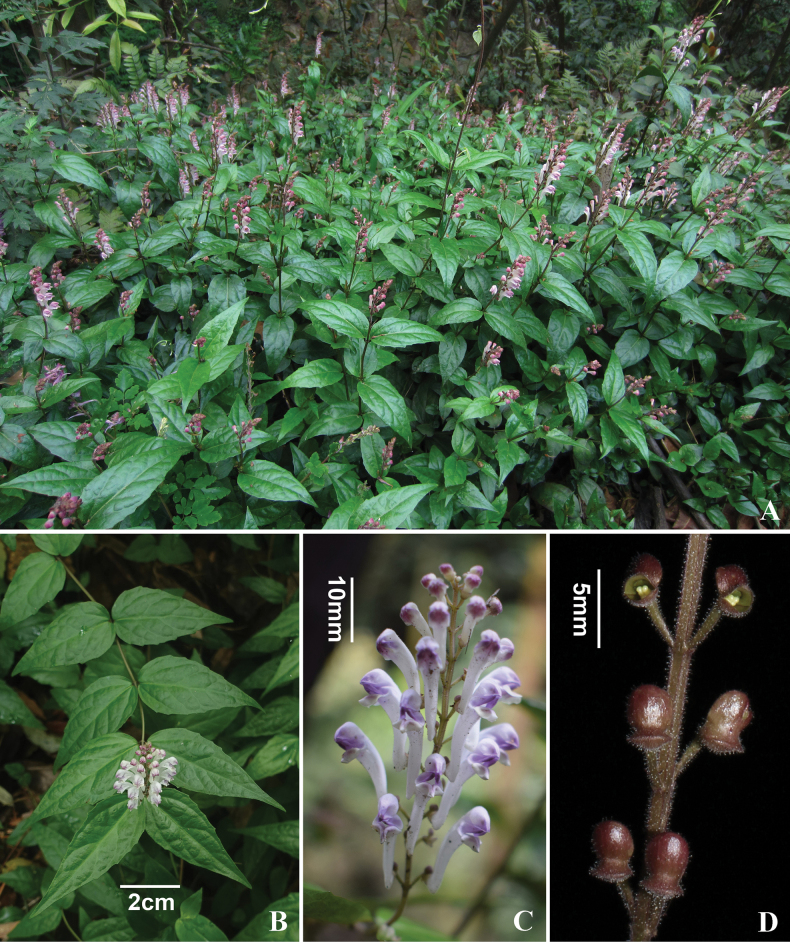
*Scutellaria tsinyunensis* C.Y.Wu & S.Chow. A. habitat; B. flowering plant; C. inflorescence; D. fruiting calyces.

#### Type.

China • Guangxi Zhuangzu Autonomous Region: Baise City (百色市), Lingyun County (凌云县), Jiayou Town (加尤镇), 24°30′N, 106°40′E, alt. ca. 950 m, 19 August 2024, *Chi Xiong et al. XC24010* (holotype IBK!; isotypes IBK!, KUN!).

#### Description.

Perennial herb. Stems 15–30 cm tall, ligneous, erect or ascending, 0.5–1.0 mm in diameter, mostly green, rarely purple or dark purple in nodes and purple pilose in stem end, glabrous downwards, unbranched or less branched. Leaves stiffly papery, lamina ovate to triangular-ovate, 14.5–23 × 8.5–14.5 mm, base broadly cuneate, rounded to truncate, margin undulate-crenate, apex obtuse to acute, glabrous on both sides, lateral veins 2–4 pairs, concave adaxially, impressed abaxially; petiole 3–7.5 mm long, dark purple, glabrous or purple pilose. Racemes terminal, 1–5 cm long, 3–10 flowers; bracts sessile, rhombic-ovate, 2–3.5 mm. Pedicel 2–3 mm long, purple pilose and sparsely glandular puberulent. Calyx ca. 2.5 mm, scutellum ca. 2 mm tall, purple pilose and sparsely glandular puberulent. Corolla tube white with purplish-red on limb, 15–18 mm long, densely white pilose and glandular puberulent outside, glabrous inside; tube bent, straight, ca. 12 mm long, base with a spur ca. 2 mm; throat ca. 4 mm wide; upper lip galeate, concave, apex emarginate, lower lip 3-lobed, median lobe triangular-ovate, ca. 6 mm wide, lateral lobes ovate, ca. 3 mm wide. Stamens 4, hidden, dimorphic; filaments flattened, ciliate above middle part; anthers densely pilose. Ovary smooth, 4-lobed; stalk short, style slender. Nutlets dark blue, ovoid ellipsoid, 1.22–1.47 × 0.79–0.98 mm, tuberculate.

#### Phenology.

Flowering from August to October; fruiting from September to November.

#### Etymology.

The specific epithet ‘*cavicola*’ refers to the cave-dwelling habit of the species. The Chinese name is given as “dòng shēng huáng qín (洞生黄芩)”.

#### Distribution and ecology.

The new species is currently known only from the type locality at an elevation of ca. 950 m. It grows in soil near the entrance of a karst cave (Fig. 2A). The most frequent co-occurring vascular species include *Achudemia boniana* (Gagnep.) L.F.Fu & Y.G.Wei, *Laportea cuspidata* (Wedd.) Friis, *Mercurialis leiocarpa* Siebold & Zucc. and *Nephrolepis cordifolia* (L.) C. Presl.

#### Additional specimens examined.

*Scutellaria tsinyunensis*: China • Chongqing Municipality, Beibei District, Jinyun Mountain, *Yangyi Group IMPCYY013* (KUN1553815!), *IMPCYY019* (KUN1550621!), same mountain, *Beibei Team 002-Y* (BJFC00110197!), same mountain, *Z.F. Xu et. al. G0005* (KUN1228330!); • Jiangjin District, Shimen Town, *Yangyi Group IMPCYY006* (KUN1554654!), Nanmuping, *Jiangjin Team 116* (SM717308028!, SM717308029!).

#### Conservation assessment.

*Scutellaria cavicola* is currently known from a single population at the type locality, consisting of approximately 100–200 mature individuals. The estimated extent of occurrence (EOO) and area of occupancy (AOO) of the new species are approximately 15,000 m^2^ and 300 m^2^, respectively. The species is found near a cave entrance on a mountainside, adjacent to a small Buddhist temple (Fig. 2A) frequented by locals for religious activities such as incense burning. Based on the IUCN Red List Categories and Criteria (IUCN 2025), the species is temporarily assessed as Critically Endangered [CR B1+2ab (ii, iii)].

## ﻿Discussion

The phylogenetic analysis revealed that, *Scutellaria cavicola*, the new species described in this study, is closely related to *S. tsinyunensis*, and together they form a clade that is sister to *S. indica* (Fig. 1). The new species displays several typical diagnostic characteristics of *Scutellaria*, including four-angled stems, opposite leaves, and prominent lobes of the scutellar epicalyx. A comparative summary of morphological traits between *S. cavicola* and *S. tsinyunensis* is provided in Table 2. The new species is readily distinguishable from *S. tsinyunensis* by its relatively short, erect, and slender woody stems triangular-ovate, stiffly papery leaves, and sparsely flowered inflorescences (Fig. 2).

To date, there are only two species of *Scutellaria* having been recorded from Karst cave habitats in China (Fu et al. 2022). The more widespread species is *Scutellaria obtusifolia* Hemsl., which is primarily distributed in cave environments across Guangxi, Yunnan, and Guizhou provinces. This species is characterised by its purple or blue-purple corolla with 2.6–3 cm in length, and can be readily distinguished from *S. cavicola*. In contrast, *S. indica* has only been found in cave habitats in Fengshan County, Guangxi. Its leaves are cordate-ovate, suborbicular to elliptical, possesses a blue corolla, further distinguishing it from *S. cavicola*.

Karst caves are considered amongst the lowest-light terrestrial habitats known for vascular plant growth (Monro et al. 2018). We noted a low proportion of flowering individuals within the population of *Scutellaria cavicola*, with most plants remaining in the vegetative stage and only a few producing inflorescences with a limited number of flowers. A similar pattern was observed in *S. obtusifolia*. In contrast, individuals growing outside the cave exhibited significantly more flowers per inflorescence than those within the cave. This could be a strategy for adapting to the low light conditions found in the cave.

## Supplementary Material

XML Treatment for Scutellaria cavicola

## ﻿Additional information

### Use of AI

No use of AI was reported.

### Author ORCIDs

Chi Xiong https://orcid.org/0000-0002-9082-2539

Zi-Yu Wei https://orcid.org/0009-0007-2393-2862

Rui-Ning Liu https://orcid.org/0009-0004-7171-2221

Feng Chen https://orcid.org/0000-0003-3398-8900

Xiao-Ying Fu https://orcid.org/0009-0009-1090-877X

Yi-Chen Wang https://orcid.org/0000-0001-9541-975X

Long-Fei Fu https://orcid.org/0000-0001-8708-4718

### Data availability

All of the data that support the findings of this study are available in the main text.
